# Rapidity in Operating

**Published:** 1907-02

**Authors:** 


### RAPIDITY IN OPERATING.

With the discoveries of anesthesia and asepsis the brilliancy and rapidity of technic cultivated by the older surgeons became for a time a minor consideration, and efforts were mostly expended in developing fields of surgery which had been previously closed. With the surety, however, that comes with larger experience, more attention is being paid to the question of technic proper. During the past year a number of papers have appeared, prominent among which may be mentioned those of Robert T. Morris and Maurice Richardson, regarding the value of rapidity in operating. The importance, in particular, of performing abdominal sections rapidly is becoming more and more recognized, and there is no question that the condition of shock is more nearly proportionate to the time consumed in operating than any other factor. That an operation should be done as rapidly as is expedient requires no argument. But it is one thing to operate rapidly and another to do so properly. The rapidity with which an operation should be performed is a very relative matter indeed. It would be manifestly unfair to sew up a typhoid perforation or to enucleate a prostate in a patient aged sixty-five, in the same ratio of time as that which we would expend in a radical amputation for carcinoma of the breast. We acknowledge the brilliancy of a surgeon who sews up a typhoid perforation in ten minutes, but we would hesitate to express our approval—at least before the classical period of three years is up— if he extirpated a cancerous breast and axillary glands with proportionate rapidity. We would prefer the extra per cent. risk of morality to that of having a half dozen cencer cells left behind in some obscure gland in the axilla. The remote as well as the immediate morality must be considered.—*Ed. Am. Jour. Surg.*, Jan. '07.

